# Cardiac Auscultation Using Smartphones: Pilot Study

**DOI:** 10.2196/mhealth.8946

**Published:** 2018-02-28

**Authors:** Si-Hyuck Kang, Byunggill Joe, Yeonyee Yoon, Goo-Yeong Cho, Insik Shin, Jung-Won Suh

**Affiliations:** ^1^ Division of Cardiology Department of Internal Medicine Seoul National University Bundang Hospital Seongnam-si Republic Of Korea; ^2^ School of Computing Korea Advanced Institute of Science and Technology Daejeon Republic Of Korea; ^3^ Division of Cardiology Cardiovascular Center Seoul National University Bundang Hospital Seongnam-si Republic Of Korea

**Keywords:** cardiac auscultation, physical examination, smartphone, mobile health care, telemedicine

## Abstract

**Background:**

Cardiac auscultation is a cost-effective, noninvasive screening tool that can provide information about cardiovascular hemodynamics and disease. However, with advances in imaging and laboratory tests, the importance of cardiac auscultation is less appreciated in clinical practice. The widespread use of smartphones provides opportunities for nonmedical expert users to perform self-examination before hospital visits.

**Objective:**

The objective of our study was to assess the feasibility of cardiac auscultation using smartphones with no add-on devices for use at the prehospital stage.

**Methods:**

We performed a pilot study of patients with normal and pathologic heart sounds. Heart sounds were recorded on the skin of the chest wall using 3 smartphones: the Samsung Galaxy S5 and Galaxy S6, and the LG G3. Recorded heart sounds were processed and classified by a diagnostic algorithm using convolutional neural networks. We assessed diagnostic accuracy, as well as sensitivity, specificity, and predictive values.

**Results:**

A total of 46 participants underwent heart sound recording. After audio file processing, 30 of 46 (65%) heart sounds were proven interpretable. Atrial fibrillation and diastolic murmur were significantly associated with failure to acquire interpretable heart sounds. The diagnostic algorithm classified the heart sounds into the correct category with high accuracy: Galaxy S5, 90% (95% CI 73%-98%); Galaxy S6, 87% (95% CI 69%-96%); and LG G3, 90% (95% CI 73%-98%). Sensitivity, specificity, positive predictive value, and negative predictive value were also acceptable for the 3 devices.

**Conclusions:**

Cardiac auscultation using smartphones was feasible. Discrimination using convolutional neural networks yielded high diagnostic accuracy. However, using the built-in microphones alone, the acquisition of reproducible and interpretable heart sounds was still a major challenge.

**Trial Registration:**

ClinicalTrials.gov NCT03273803; https://clinicaltrials.gov/ct2/show/NCT03273803 (Archived by WebCite at http://www.webcitation.org/6x6g1fHIu)

## Introduction

Cardiovascular diseases are the most common causes of death, accounting for 31.5% of all deaths globally [1,2]. In 2015, in the United States, 92.1 million adults were estimated to have cardiovascular diseases, and 43.9% of the adult population is projected to have some form of cardiovascular disease by 2030 [3].

The stethoscope has played a key role in the physical examination of patients with cardiac disease since its invention by Rene Laënnec in 1816 [4]. The opening and closing of the heart valves, as well as blood flow and turbulence through the valves or intracardiac defects, generate rhythmic vibrations, which can be heard via the stethoscope [5]. Cardiac auscultation using the stethoscope enables hemodynamic assessment of the heart and can help in the diagnosis of cardiovascular diseases [6].

Recently, the advent of noninvasive imaging modalities has dwarfed the importance of cardiac auscultation in clinical practice [7,8]. Devices such as the handheld ultrasound have enabled detailed on-site visualization of the cardiac anatomy and are further threatening the role of the stethoscope as a bedside examination tool [9,10]. In this way, there has been a decrease in the appreciation of the importance of cardiac auscultation, and physicians are decreasingly proficient and confident in their examination skills [11-13]. Studies have also suggested a low level of interobserver agreement regarding cardiac murmurs [14].

The smartphone has become a popular device. As of 2015, 64% of Americans and 88% of South Koreans were reported to own a smartphone [15]. Smartphones are frequently used for health purposes, such as counseling or information searches [16]. The modern smartphone has excellent processing capability and is equipped with multiple high-quality components, such as microphones, display screens, and sound speakers. There have been efforts to use smartphone health apps for self-diagnosis [17]. However, some of these software apps have shown poor credibility, and their role in health care is not yet established [18].

Therefore, we sought to develop a smartphone app for cardiac auscultation that could be used by non–medical expert users. Although the importance of cardiac auscultation is declining in the hospital setting, it could serve as a screening tool at the prehospital stage if it can be performed easily by smartphone users themselves. This was a pilot study to test the feasibility of cardiac auscultation using the built-in microphones of smartphones without any add-on devices. The study tested (1) whether heart sound recording using a smartphone is feasible, and (2) whether an automated diagnostic algorithm can classify heart sounds with acceptable accuracy. Heart sounds were recorded using the smartphone microphones and processed electronically. We developed a diagnostic algorithm by applying convolutional neural networks, which we used for the diagnosis of the recorded heart sounds. In this study, we assessed the diagnostic accuracy of the algorithm.

## Methods

### Description of the App

**Figure 1:**
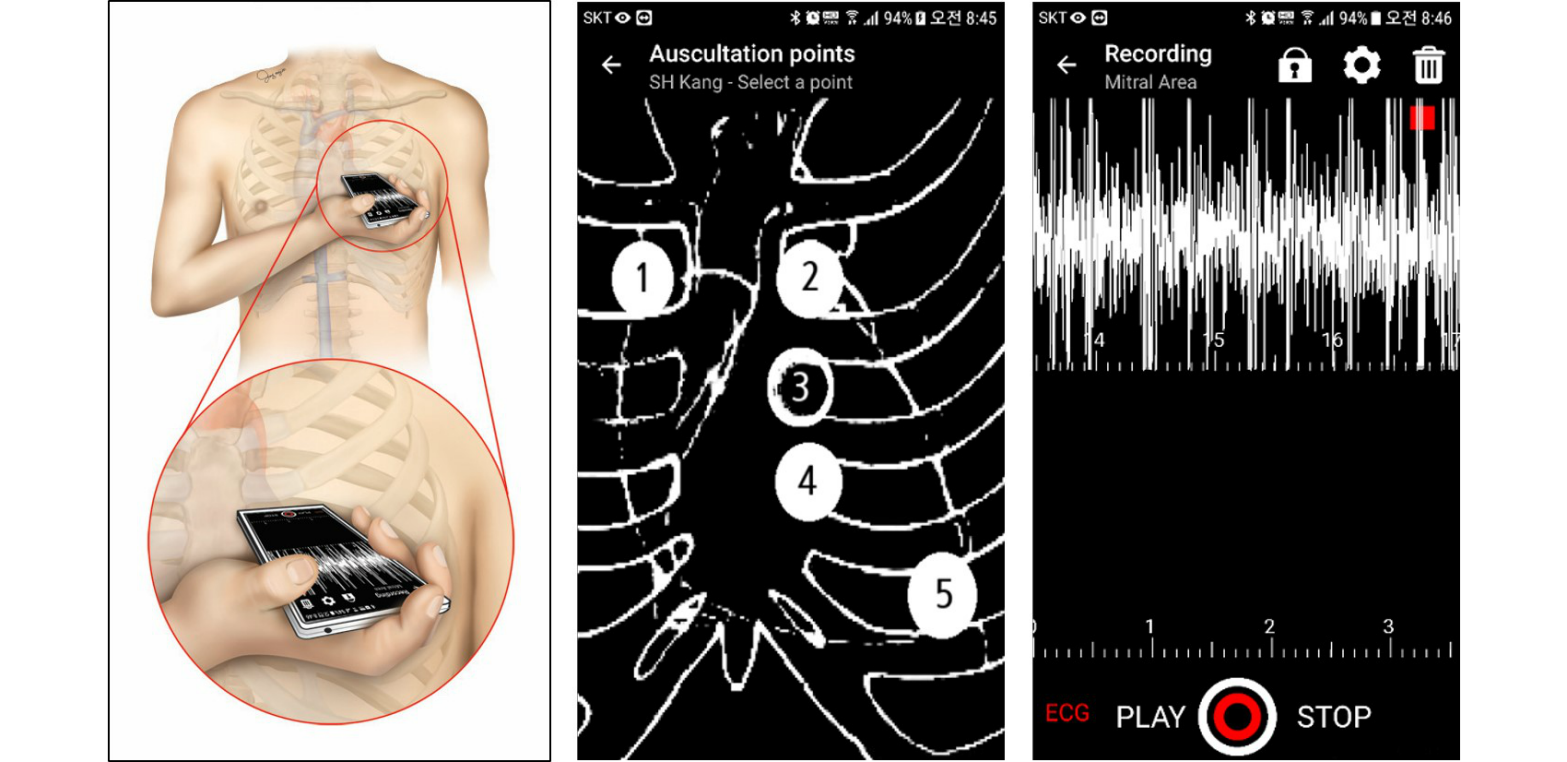
Heart sound recording using a smartphone app. Left: illustration of how the heart sounds were recorded in this study. Smartphones were placed directly on the chest wall; a dedicated app was used with no add-on devices. Middle and right: representative screenshots of the app (called CPstethoscope) developed for this study. ECG: electrocardiogram.

We developed a smartphone app named CPstethoscope for this study (Figure 1). The app runs on the Android operating system (Google Inc) and is used for research purposes only. Heart sounds were recorded by placing the phone on the skin of the chest, using the built-in microphone. In most smartphones, microphones are located on the lower border of the device. Heart sounds can be best heard in the intercostal spaces. The instructions for this app indicated the anatomical landmarks and auscultation areas. While maintaining the contact of the lower margin of the smartphone with the chest wall, users are required to manipulate the device to start and stop recording. Users can see on the screen whether their heart sounds are properly being captured.

### Study Design

This was a pilot study designed to demonstrate the feasibility of smartphone-based recording and identification of heart sounds. We sought to enroll 50 participants who were 18 years of age or older and had undergone an electrocardiogram (ECG) and echocardiography within the previous 6 months at Seoul National University Bundang Hospital, Seoul, Republic of Korea. Ultimately, we sought to develop an app for self-diagnosis that could be performed by users. However, for this pilot study, heart sounds were recorded by researchers who were familiar with the use of the app and understood the principles of cardiac auscultation. The investigators who recorded heart sounds were not aware of the patients’ diagnoses. Eligible patients were invited to participate in the study by the research doctors at the outpatient clinics or on the wards. After participants provided informed consent, their heart sounds were recorded in a quiet room that was free from environmental noises.

Reference heart sounds were recorded by participating cardiologists (SHK, YY, GYC, and JWS) using an electronic stethoscope (3M Littmann Electronic Stethoscope Model 3200; 3M, St Paul, MN, USA). Study devices were the Samsung Galaxy S5 (model SM-G900) and Galaxy S6 (SM-G920; Samsung Electronics, Suwon, Republic of Korea), and LG G3 (LG-F400; LG Electronics, Seoul, Republic of Korea).

We chose the best site for recording from among the aortic, pulmonic, mitral, and tricuspid areas. The built-in microphones were placed directly on the skin of the chest wall for detection of the heart sound. We tested all 3 devices with all study participants. There were no prespecified orders for tested devices. No add-on devices were used. Recordings were made for approximately 10 seconds after stable heart sounds were displayed on the screen. Final diagnoses of the reference heart sounds were confirmed by a second cardiologist (SHK and GYC) by listening to the audio files and matching them with the echocardiography reports.

This study was approved by the Seoul National University Bundang Hospital institutional review board on August 24, 2016 (B-1609-361-303), and all participants provided written informed consent. We registered this study protocol (ClinicalTrials.gov NCT03273803). The corresponding author had full access to all the data in the study and takes responsibility for its integrity and the data analysis.

### Data Processing and Identification

We transferred the recorded audio files to a desktop computer for data processing. After subtracting environmental and thermal noises using fast Fourier transformation, we constructed time-domain noise-reduced heart sounds. We detected the first and second heart sounds without an ECG reference, using a previously reported algorithm [19]. Time-domain signals were transformed into frequency-domain spectrogram features. We developed a diagnostic algorithm using convolutional neural networks, a variant of an artificial neural network that mimics the connections of neurons in the visual cortex of the brain. The convolutional neural network was constructed from 40×40 heart sound spectrogram matrices through 1 input layer. We processed these matrices with 2 convolution-max pool layers whose kernel size was 5×5. Moreover, the number of kernels for each of the 2 convolutional layers was either 8 or 16. Next, a dense, fully connected layer followed the second convolution–max pool layer, and we appended the last readout layer with 5 nodes that corresponded to each disease. We calculated the training loss of function of the network as soft maximum cross-entropy using the values from the readout layer. Finally, we trained the network with the Adam optimizer at a learning rate of 0.001 [20]. We used the TensorFlow version 1.2 Python library to compose this network [21]. Training was conducted using demonstration heart sounds obtained from open databases (The Auscultation Assistant, C Wilkes, University of California, Los Angeles, Los Angeles, CA, USA; Heart Sound & Murmur Library, University of Michigan Medical School, Ann Arbor, MI, USA; Easy auscultation, MedEdu LLC, Westborough, MA, USA; 50 Heart and Lung Sounds Library, 3M, St Paul, MN, USA; and Teaching Heart Auscultation to Health Professionals, J Moore, Rady Children’s Hospital, San Diego, CA, USA). We classified heart sounds into 5 categories: normal, third heart sound, fourth heart sound (S_4_), systolic murmur, and diastolic murmur. The algorithm showed 81% diagnostic accuracy with the training sets. Testing was performed with the samples acquired from this study.

### Statistical Analysis

We calculated continuous variables as mean (SD), and categorical variables as counts and percentages. Reference heart sounds were adjudicated by experienced cardiologists. The primary end point of the study was the diagnostic accuracy of the system for heart sound classification. We considered the diagnosis to be accurate when the algorithm classified a heart sound into the correct category with 50% or more probability. We also estimated the performance of the system using sensitivity, specificity, positive predictive value, and negative predictive value. We defined the study end points were as follows: diagnostic accuracy = (TP+TN)/(TP+FP+FN+TN); sensitivity = TP/(TP+FN); specificity = TN/(TN+FP); positive predictive value = TP/(TP+FP); and negative predictive value = TN/(TN+FN), where TP indicates true positive; TN, true negative; FP, false positive; and FN, false negative. We calculated the diagnostic values as simple proportions with corresponding 95% confidence intervals. Statistical analyses were performed using the R programming language version 3.2.4 (The R Foundation for Statistical Computing). A 2-sided *P*<.05 was considered statistically significant.

## Results

### Patient Profiles

A total of 46 patients participated in this study. Multimedia Appendix 1 shows the Standards for Reporting of Diagnostic Accuracy Studies checklist and flow diagram for the study. Similar numbers of men and women were enrolled, and their median age was 65.5 years. Table 1 describes the participants’ characteristics: 20 (44%) had systolic murmurs, 20 (44%) had normal heart sounds, 5 (11%) had diastolic murmurs, and 1 (2%) had S_4_.

After audio file processing, including noise reduction, we confirmed 30 of 46 heart sounds (65%) as interpretable. The reasons for failure to acquire interpretable heart sounds included the small amplitude of the acquired heart sounds, background noise, and the participant’s poor cooperation. Younger age tended to be associated with better interpretability, while body mass index had no impact. Significant factors for uninterpretability included atrial fibrillation and diastolic murmur.

### Diagnostic Performance

**Figure 2:**
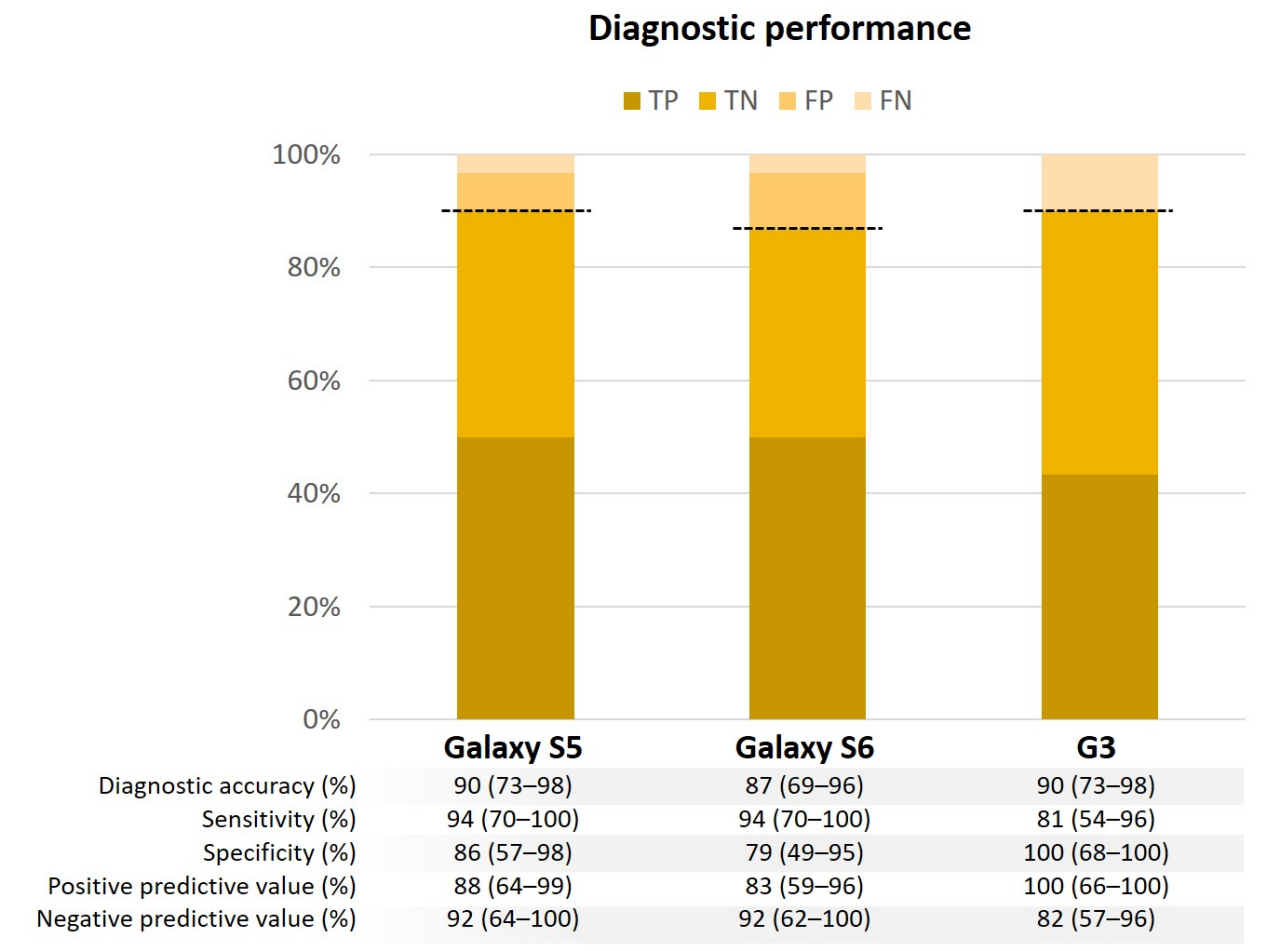
Diagnostic performance of each study device. Bold broken lines indicate diagnostic accuracy. FN: false negative; FP: false positive; TN: true negative; TP: true positive.

**Figure 3:**
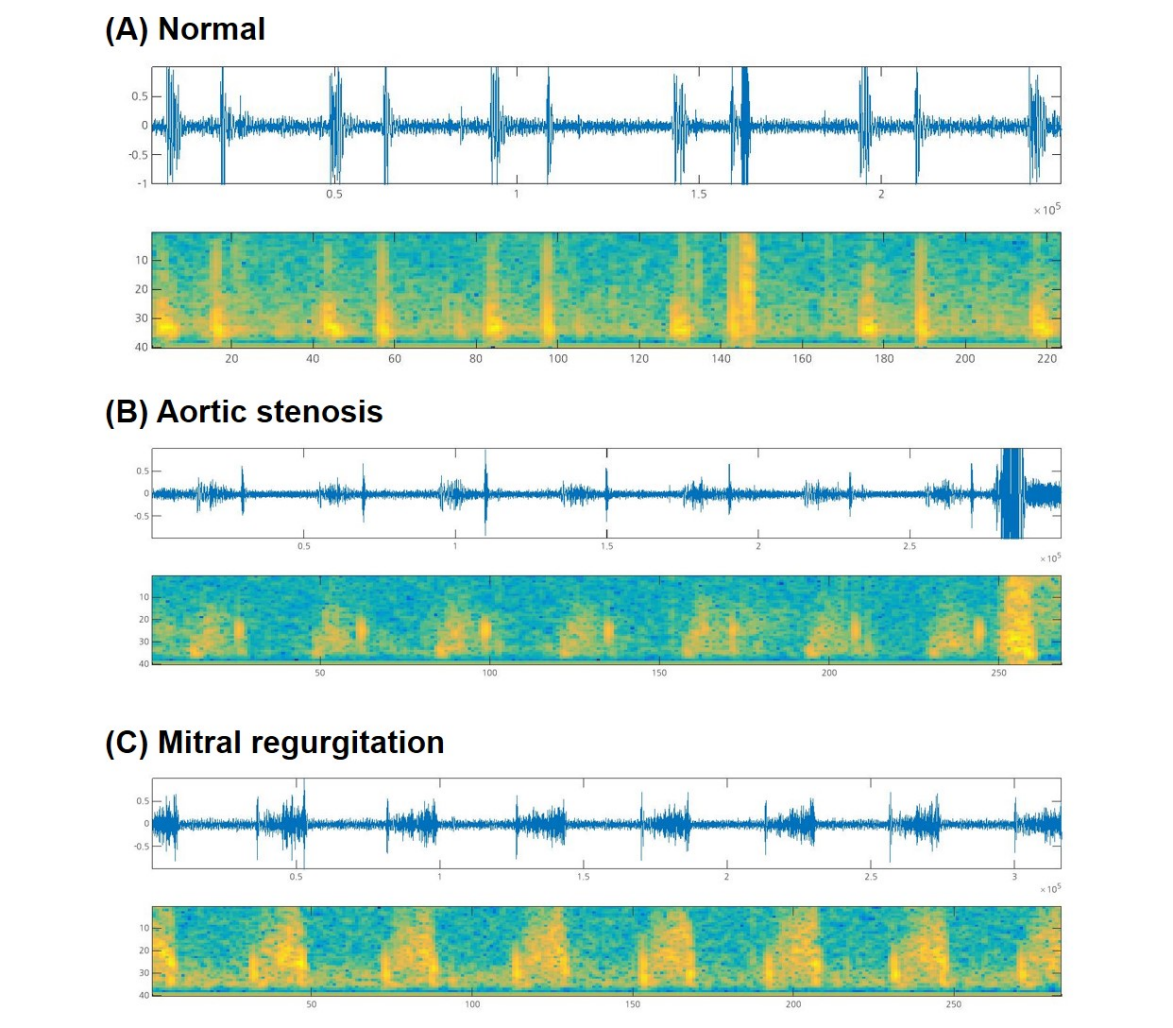
Representative phonocardiograms and spectrograms. (A) Normal heart sounds from the aortic area of a 22-year-old man with a history of vasovagal syncope. (B) Midsystolic ejection murmur from the aortic area of an 83-year-old woman with aortic stenosis, which was classified as a systolic murmur. (C) Systolic murmur from the mitral area of a 63-year-old woman with mitral valve prolapse and mitral regurgitation, which was classified as a systolic murmur.

Figure 2 shows the performance of the diagnostic algorithm by device. Heart sounds recorded with the 3 different study devices yielded consistently high diagnostic accuracy: Samsung Galaxy S5, 90% (95% CI 73%-98%); Samsung Galaxy S6, 87% (95% CI 69%-96%); and LG G3, 90% (95% CI 73%-98%). The Samsung Galaxy S5 and S6 showed a high sensitivity (S5: 94%, 95% CI 70%-100%; S6: 94%, 95% CI 70%-100%), while the LG G3 showed a high specificity (100%, 95% CI 68%-100%). The diagnostic performance did not vary significantly according to the study participants’ age or sex (Table 2). Figure 3 shows representative waveforms and spectrograms of heart sounds (audio files are provided in Multimedia Appendix 2,Multimedia Appendix 3, and Multimedia Appendix 4). No meaningful adverse events occurred during the study.

**Table 1:** Characteristics of study participants.

Characteristics		Interpretable heart sounds		Total	*P* value^a^
		Yes	No		
Number of participants, n (%)		30 (65)	16 (35)	46	
Male sex, n (%)		14 (47)	7 (44)	21 (46)	>.99
Age (years), median (range)		62.5 (22.0-90.0)	72.0 (27.0-88.0)	65.5 (22.0-90.0)	.07
Body mass index (kg/m^2^), mean (SD)		23.8 (3.9)	23.4 (3.3)	23.7 (3.7)	.69
Hypertension, n (%)		14 (47)	9 (56)	23 (50)	.76
Diabetes, n (%)		4 (13)	5 (31)	9 (20)	.24
Atrial fibrillation, n (%)		0 (0)	5 (31)	5 (11)	<.001
**Primary diagnosis, n (%)**					.005
	Aortic stenosis	11 (37)	2 (13)	13 (28)	
	Aortic regurgitation	0 (0)	2 (13)	2 (4)	
	Mitral stenosis	0 (0)	4 (25)	4 (9)	
	Mitral regurgitation	2 (7)	0 (0)	2 (4)	
	Hypertrophic cardiomyopathy	1 (3)	1 (6)	2 (4)	
	Others	16 (53)	7 (44)	23 (50)	
**Heart sounds, n (%)**					.007
	Systolic murmur	15 (50)	5 (31)	20 (44)	
	Diastolic murmur	0 (0)	5 (31)	5 (11)	
	S_3_/S_4_^b^	1 (2)	0 (0)	1 (2)	
	Normal	14 (47)	6 (38)	20 (44)	

^a^Comparisons were performed using Student *t* test or Mann-Whitney *U* test for continuous variables, and chi-square test or Fisher exact test for categorical variables.

^b^S_3_/S_4_: third and fourth heart sounds.

**Table 2:** Diagnostic performance (%) of each study device.

Participants grouped by age and sex		Study device		
		Galaxy S5 estimate (95% CI)	Galaxy S6 estimate (95% CI)	G3 estimate (95% CI)
**Total (n=30)**				
	Diagnostic accuracy	90 (73-98)	87 (69-96)	90 (73-98)
	Sensitivity	94 (70-100)	94 (70-100)	81 (54-96)
	Specificity	86 (57-98)	79 (49-95)	100 (68-100)
	Positive predictive value	88 (64-99)	83 (59-96)	100 (66-100)
	Negative predictive value	92 (64-100)	92 (62-100)	82 (57-96)
**Men (n=14)**				
	Diagnostic accuracy	79 (49-95)	79 (49-95)	93 (66-100)
	Sensitivity	83 (36-100)	83 (36-100)	83 (36-100)
	Specificity	75 (35-97)	75 (35-97)	100 (52-100)
	Positive predictive value	71 (29-96)	71 (29-96)	100 (36-100)
	Negative predictive value	86 (42-100)	86 (42-100)	89 (52-100)
**Women (n=16)**				
	Diagnostic accuracy	100 (71-100)	94 (70-100)	88 (62-98)
	Sensitivity	100 (59-100)	100 (59-100)	80 (44-97)
	Specificity	100 (42-100)	83 (36-100)	100 (42-100)
	Positive predictive value	100 (59-100)	91 (59-100)	100 (52-100)
	Negative predictive value	100 (42-100)	100 (36-100)	75 (35-97)
**Elderly (≥65 years; n=12)**				
	Diagnostic accuracy	100 (64-100)	92 (62-100)	83 (52-98)
	Sensitivity	100 (55-100)	100 (55-100)	78 (40-97)
	Specificity	100 (19-100)	67 (9-99)	100 (19-100)
	Positive predictive value	100 (55-100)	90 (55-100)	100 (47-100)
	Negative predictive value	100 (19-100)	100 (9-100)	60 (15-95)
**Young (<65 years; n=18)**				
	Diagnostic accuracy	83 (59-96)	83 (59-96)	94 (73-100)
	Sensitivity	86 (42-100)	86 (42-100)	86 (42-100)
	Specificity	82 (48-98)	82 (48-98)	100 (62-100)
	Positive predictive value	75 (35-97)	75 (35-97)	100 (42-100)
	Negative predictive value	90 (55-100)	90 (55-100)	92 (62-100)

## Discussion

### Principal Findings

This was a pilot study to assess the feasibility of heart sound recording and identification using smartphones. We found that reliable heart sound recording was the most important difficulty encountered. However, the results of this study suggest that, once interpretable heart sounds are acquired, cardiac murmur diagnosis using convolutional neural networks yields high diagnostic accuracy.

### Implications

With the widespread use of smartphones, an increasing number of health care apps have been developed. There were approximately 165,000 health-related apps available in 2016 [22]. These health care-related apps comprise a variety of aspects of medicine, including prevention, diagnosis, monitoring, treatment, compensation, and investigation [23]. However, there are concerns that many of these apps are not evidence based, and it is difficult to find any information on the research used in their development [18]. This study was a part of our effort to develop a diagnostic app that can differentiate normal and abnormal heart sounds. We sought to validate the diagnostic performance of the recording and identification system among patients in real-world clinical practice.

There have been attempts to use add-on gadgets in conjunction with smartphones for health care use, but most of these have not been accepted widely. Modern smartphones are equipped with high-quality built-in microphones that can capture low-pitch, low-amplitude heart sounds. We presumed that an app working solely with the featured specifications would have advantages with respect to accessibility and acceptability. However, this study implied that the acquisition of good-quality heart sounds is still far from perfect. A variety of factors were suggested to affect the heart sound recording. First, background noise is difficult to reduce systematically and, thus, should be avoided during recordings. It was necessary to record on the skin of the chest wall, and the choice of the appropriate location was essential. Second, respiration was not as important as expected. The frequency spectrum of lung sounds (100-2500 Hz) is usually distant from that of heart sounds (20-100 Hz) [24]. Thus, lung sounds were easily attenuated by applying a simple band-pass filter. Third, patient factors, such as age, body mass index, and the presence of arrhythmia, were also crucial. Fourth, our system failed to recognize heart sounds with diastolic murmur, although the sample size was small.

The use of machine learning in clinical medicine is rapidly increasing, with a marked increase in the amount of available data [25]. The interpretation of digitized images and development of prediction models are the leading applications of machine learning in the field of medicine [26,27]. This study suggests that the interpretation of audio signals derived from humans may be a potential application of artificial intelligence.

### Comparison With Prior Work

To our knowledge, this study is the first attempt to discriminate heart sounds using a deep learning–based diagnostic algorithm. We showed that the diagnostic algorithm was feasible and reproducible. We found only 1 app for cardiac auscultation that enables heart sound recording, which is called the iStethoscope [28]. It amplifies and filters heart sounds in real time for better quality, but it is not capable of diagnosing heart murmurs. AliveCor Kardia, a device approved by the US Food and Drug Administration, enables ECG monitoring and, according to 1 clinical trial, significantly improves the detection of incident atrial fibrillation [29]. Azimpour et al performed an elegant study in which they used an electronic stethoscope to detect stenosis of coronary arteries [30]. Although the study idea was interesting, it may be difficult to use in commonly available smartphones due to the deep location of the coronary arteries and the low amplitude of the acoustic signals. There are several apps that enable heart rate monitoring. Some require specialized devices, and others simply use built-in smartphone cameras and flashes, also known as photoplethysmography. However, their accuracy and clinical application still require further investigation [31,32].

### Limitations

This study had several limitations. First, the sample size was too small to represent a variety of cardiac murmurs. Second, the enrollment of study participants was selective rather than consecutive; thus, there is a possibility of a selection bias of participants with clear and unambiguous heart sounds. Third, we used the app developed for this study only to record heart sounds. In this pilot study, the audio files were moved to a central server and subsequently analyzed. Therefore, the app needs to be improved such that it can be used in the real world, such as an all-in-one system from acquisition to diagnosis. Fourth, we obtained the heart sounds ourselves, although we ultimately seek to develop an app that can be used by members of the general population.

Fifth, this study showed variations in performance with different devices, which seem to be caused by the differing specifications of each smartphone. This is one of the major hurdles in the development of an app that can be used in a variety of smartphones from different manufacturers. Our pilot testing indicated that the quality of recorded heart sounds depended on the quality of the built-in microphones. For this reason, we included 3 high-end smartphones for this study. System performance may be worse with inexpensive devices. In addition, we tested only devices running the Android operating system in this study, but not the Apple iPhone, which is one of the most widely used smartphones worldwide.

### Future Research Steps

The app described in this study requires further development. An all-in-one system is crucial, comprising recording, audio processing, and a diagnostic algorithm. Instructions that help users record their heart sound by themselves are also needed. We are improving the ability of the app to acquire interpretable heart sounds and to diagnose atrial fibrillation. Another potential application is the use of a diagnostic algorithm with commercialized electronic stethoscopes performed by medical personnel [33]. This may improve the quality of clinical practice by assisting early-career doctors or nurses to assess patients.

### Conclusions

The concept of cardiac auscultation using smartphones is feasible. Indeed, diagnosis using convolutional neural networks yielded a high diagnostic accuracy. However, use of the built-in microphones alone was limited in terms of reproducible acquisition of interpretable heart sounds.

## Appendix

Multimedia Appendix 1 Standards for Reporting of Diagnostic Accuracy Studies study checklist and study diagram.

Multimedia Appendix 2 Audio file 1 (normal heart sound).

Multimedia Appendix 3 Audio file 2 (aortic stenosis).

Multimedia Appendix 4 Audio file 3 (mitral regurgitation).

## Abbreviations

ECG: electrocardiogram
FN: false negative
FP: false positive
S4: fourth heart sound
TN: true negative
TP: true positive
